# Health promotion networks in two districts in Bavaria, Germany: an exploratory case study mapping networks with respect to thematic agenda and location

**DOI:** 10.3389/fpubh.2023.1111642

**Published:** 2023-06-27

**Authors:** Annika Marie Fehrmann, Kathrin Steinbeisser, Andrea R. Wolff, Michaela Coenen

**Affiliations:** ^1^Institute for Medical Information Processing, Biometry, and Epidemiology – IBE, Chair for Public Health and Health Services Research, Faculty of Medicine, LMU Munich, Munich, Germany; ^2^Pettenkofer School of Public Health, Munich, Germany; ^3^Coordinating Office for Health Equity, Bavarian Association for Health Promotion and Disease Prevention, Munich, Germany; ^4^Faculty for Applied Healthcare Sciences, Deggendorf Institute of Technology, Deggendorf, Germany

**Keywords:** health promotion, health equity, network, network mapping, geographical mapping, agenda mapping, strategic networking, network development

## Abstract

**Introduction:**

Building networks is an essential part of health promotion. However, network analysis remains relatively unexplored in this field. This study introduces a new technique that maps thematic agendas and geographical locations of health promotion actors.

**Methods:**

This case study used elements of quantitative and qualitative methods to analyse network data. We used empirical data from two networks in Bavaria, a federal state of Germany.

**Results:**

We identified a total of 55 actors in the first network and 64 actors in the second. We categorized the thematic agenda of actors according to their main field of work: “healthy childhood development,” “healthy middle age phase,” “healthy ageing,” “health equity in all phases of life.” One network showed a significant surplus of actors that focus on “healthy ageing.” We combined and analysed data from both networks collectively. Two districts with no health promotion actors within their geographical borders were identified. To put geographical gaps into context, data about deprivation and age was included.

**Discussion:**

Results identified geographical areas with high need for support from health promotion actors. Through comparison of our results with existing literature, we derived potential network strategies for further successful networking. This study adds a new perspective to characterize health promotion networks by mapping them thematically and geographically. The concept can be used to give health promotion organisations relevant insight into network structures. This can improve decision-making processes concerning partnership strategy and finally lead to a positive health impact. Hence, our findings encourage further development of this technique and other networking methods in the field of health equity and health promotion.

## 1. Introduction

In 1996, the World Health Organisation (WHO) acknowledged networking as an important pillar of achieving health for all (1). Networking can improve equal access to health-promoting activities, encourage participation (e.g., in dialogue), and develop intersectoral action by making resources for health promotion easily available (1). Hence, building networks is defined as “a key way of working” in health promotion (2). This matches the idea that partnership processes produce synergies such as having access to complementary skills and perspectives, sharing work, common feelings of excitement, and effective problem-solving (3). Thus, actors involved in health promotion strategies and activities can achieve better outcomes when working together than when working alone (3).

Building networks requires sound planning of structures, strategies, and interventions (4). However, health promotion strategies often lack such a conceptual basis. This complicates network analysis (4). The most common method of mapping and analysing networks in health systems, and more specifically health promotion activities, is the so-called “Social Network Analysis” (SNA) (5). SNA measures relationships among actors and assesses factors that influence a network’s structure (6). The network is seen as a set of actors and relationships among them, and most social network studies include attribute data that indicate actors or these relationships (6). However, not all attributes which provide valuable information for comprehensively describing the state of a network are assessed and integrated into an SNA.

In particular, attributes related to individual or institutional goals are critical for successful partnership processes but seem to be neglected in network analysis (7). Researchers studying the development of health policies in Dutch municipalities found that actors become part of a network when it supports their organisational domains and needs, not just for the sake of general health issues (8). Therefore, actors’ thematic agendas, which we define as the actors’ goal that they pursue through the help of a network, should be considered with respect to network analysis.

Another important attribute to consider in network analysis is the geographical location of actors. The WHO advises organisations to adapt health promotion strategies to local needs (9). Geographical representation of network data can give insight into neglected areas and opportunities for additional outreach (10). Accordingly, it is reasonable to identify geographical areas in need for stronger support in terms of health promotion actors and programs. To precisely identify such areas, regional data on sociodemographic characteristics should be further considered. One strategy could be to include data regarding deprivation. Jarman et al. (11) emphasized the importance of concentrating health resources in deprived, underprivileged areas. Additionally, age patterns influence whether geographical areas are in need. This can potentially indicate which age group needs the most support in a certain geographical area.

Given the state of the evidence, the overall objective of this study is to conduct a case study aiming to explore two health promotion networks coordinated by an institution (Coordinating Office for Health Equity in Bavaria, KGC)^1^ in Germany. Furthermore, we examine whether existing actors actually address the needs of the targeted population. The specific aims of this study are:

1. To identify health promotion actors among two networks linked to the KGC;
2. To explore the strength of relationships between health promotion actors and the KGC;
3. To identify the actors’ geographical location and their thematic agenda;
4. To analyse thematic cliques, geographical clusters, and gaps in the networks;
5. To illustrate the context in which actors operate by analysing associations between the geographical distribution of actors and their thematic agenda, deprivation, and age.

## 2. Methods

### 2.1. Study design

This is a case study design using SNA principles of assessing actors and factors that influence a network’s structure by applying elements of both quantitative and qualitative methods. We collected empirical data based on datasets and documents from two networks in Bavaria, a federal state in Germany. Those two networks are coordinated by the Coordinating Office for Health Equity in Bavaria (KGC, *German: Koordinierungsstelle Gesundheitliche Chancengleichheit*). The KGC is a health promotion organisation and part of the Germany-wide cooperation network “Equity in Health,” which was established by the Federal Center for Health Education *(German: Bundeszentrale für gesundheitliche Aufklärung)* in 2002 (12). KGCs exist in all German federal states and supports the community partner process “HEALTH FOR ALL”; it strengthens a collaborative learning process and professional exchange to promote the health of people of all ages across social and health sectors (12). Therefore, KGCs consult and support relevant actors in health promotion. They form and strengthen networks between such actors to encourage sustainable development (12). Hereafter, “KGC” refers to the Bavarian KGC. To foster effective networks, the KGC organises events and activities where actors can meet and work together to promote health and health equity. Thus, the KGC serves as a central actor that identifies and connects actors working in health promotion. For this study, we selected two Bavarian networks: Upper Palatinate (approximately 1.1 million residents in 2020) and Lower Bavaria (approximately 1.2 million residents), located in eastern Bavaria on the borders of the Czech Republic and Austria (delimited by official geographical definitions of the Bavarian administrative districts) (13). These networks were targeted because the KGC has built stable networks and connections between actors there. Bavaria is, with a surface area of 70.500 km^2^, the largest German federal state, has more than 13 million inhabitants, and consists of seven administrative regions (13, 14).

### 2.2. Variables

An “actor” in our study is defined as an individual who is currently or has been in contact with the KGC (e.g., a member of a health promoting club or association, a public official, a volunteer). An actor’s geographical location was operationalized by using postal codes (an established proxy for addresses (15)). We defined the thematic agendas based on pre-existing categories according to the Bavarian Prevention Guideline (16). The guideline specifies the following categories of health promotion activities (16): “healthy childhood development,” “healthy middle age phase,” “healthy ageing,” and “health equity in all phases of life” (16).

The strength of a relationship (“tie” in networking terms) between actors and the KGC was estimated by measuring the frequency of contact within the last 24 months (17). The following answer options were adapted based on established methods (18): “no contact,” “once in 24 months,” “yearly,” “semi-annually,” “quarterly,” “monthly,” “weekly,” and “daily.”

We used the Bavarian Index of Multiple Deprivation (BIMD) as a measure of deprivation at the district level. The BIMD is a measure of relative deprivation which combines indicators in certain domains of deprivation to generate a comprehensive deprivation index (19, 20). The index, last updated in 2015, consists of the following seven domains: income (weight: 25%), employment (25%), education (15%), district revenue (15%), social capital (10%), environment (5%), and security (5%) (19, 21). High BIMD values indicate high deprivation (meaning low income, high unemployment, etc.) compared to nearby districts. Originally, the BIMD was created by Maier et al. (19) who calculated deprivation scores for Bavarian communities. BIMD values are often represented in quintiles (e.g., quintile 1: least deprived, quintile 5: most deprived) (21). In this study, we used the overall score for each district instead of quintiles because we wanted to compare the scores between districts. The scores were provided by W. Maier, originator of the BIMD (19). We included the ageing quotients and youth ratios published by the Bavarian Statistical Office (13). The Bavarian Statistical Office calculates these metrics on a regular basis (13). For this study, we used the ageing quotient and youth ratio for the year 2019 and an predicted ageing quotient and youth ratio for the year 2039 (13). We gathered the data from their official website. The Bavarian Statistical Office operationalizes the ageing quotient as the number of people aged 65+ per 100 people aged 20 to 64 (13). The youth ratio is operationalized as the number of people aged 0 to 19 per 100 people aged 20 to 64 (13).

### 2.3. Data collection and preparation for Lower Bavaria

In contrast to Upper Palatinate, no network data existed for Lower Bavaria. Therefore, we collected data during this study with the following process. We analysed original datasets and documents from the KGC. Originally, this data was documented for the planning and documentation of events and meetings. Now, we focused on the network variables mentioned above. We used a structuring content analysis (22) to examine 17 documents consisting of programs, protocols, event announcements and summaries, articles from local newspapers, registration and participant lists, flyers and invitations, one follow-up survey, correspondence, one counselling list, and articles from the official website.^2^ Available documents existed for the years 2017 to 2021. Event articles were from the years 2012 to 2021. Documents had been included if the actors (“nodes” in network terms (6)) were mentioned in the texts and were located in Lower Bavaria. Thus, actors were identified retrospectively.

**Figure 1:**
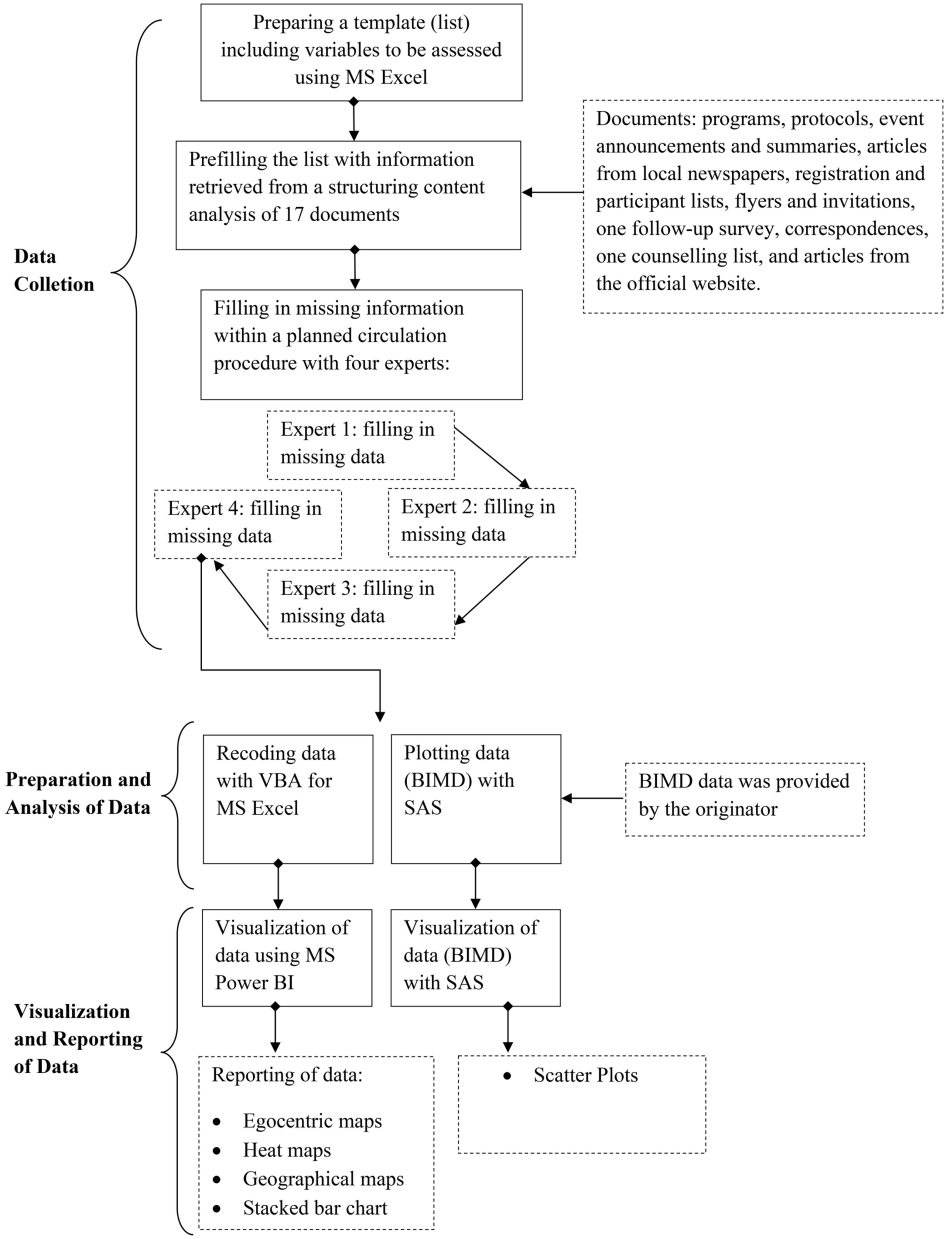
Steps of data collection and preparation.

We generated an MS Excel list of actors and network-related variables. One KGC expert pretested the list for comprehensibility and gave the researchers feedback. In a next step, we asked experts from the KGCs to list their number of contacts with actors within the last 24 months, which we used to measure the frequency of contact, in accordance with established research methods (17). Four KGC experts filled in missing information and corrected false codification during a planned, two-week circulation procedure. We had recruited the experts previously via e-mail in April 2021 and had sent them a detailed instructional video on how to fill in data correctly. The list’s structure allowed tracking the person who filled in information for remaining questions. In a final step, we asked experts to name actors that had not been listed. The data was then prepared as described in the data analysis. The process of data collection and preparation is visualized in Figure 1.

### 2.4. Data preparation for Upper Palatinate

We used pre-existing network data from Upper Palatinate. The data had been gathered by the KGC itself. The KGC collected the data a year before data was collected for this study. The data collection process was similar to the one used in this study. Data was collected within a content analysis study and an expert survey study. The dataset also included the variables “actor,” “geographical location,” and “thematic agenda,” which were operationalized equally. The “frequency of contact” was also measured, but operationalized differently. It was measured as the frequency of contact within the last 12 months (compared to this study: frequency of contact within the last 24 months). As the COVID-19 pandemic naturally decreased the number of contacts during the last months in this period, we chose the longer time-span in order to mitigate the effects on our observations. We excluded further variables from data for Upper Palatinate since they did not concern our research objective. We recoded data using Visual Basic Applications for MS Excel (Excel version 16.0).

### 2.5. Data analysis

We visualised data about actors through egocentric maps using Microsoft Power BI (version 2.88.1144.0). We excluded actors if they were located outside Upper Palatinate or Lower Bavaria, or if the frequency of contact was missing.

To explore the strength of a tie, we dichotomised responses regarding “frequency of contact” consistent with existing methods in network analysis (18). “Having contact at least quarterly” was considered a strong tie; “having contact semi-annually or less” was considered a weak tie. When experts chose “no contact,” actors were marked as “inactive.” The tie strength was also visualised using egocentric maps.

To identify the actors’ geographical location, we used ArcGIS Maps for Power BI to create geographical maps based on postal codes. Since some actors had the same postal code, we calculated a new weighted variable to display geographically overlapping actors. Actors were visualised in geographical maps using points adjusted in size according to the new weighted variable, and in heat maps.

To identify the actors’ thematic agenda, we examined the categories (“healthy childhood development,” “healthy middle age phase,” “healthy ageing,” “health equity in all phases of life”) by applying the principles of structuring content analysis according to Mayring (22). The method allows for the extraction of relevant content based on predefined categories (22). At the same time, those predefined categories can be adapted, if necessary (22). Prototypical text passages, definitions, and coding rules can be extracted during the analysis (22). A coding manual allowed us to assign actors to categories. Experts also assigned actors to thematic categories. The interrater reliability of the experts was calculated using Cohen’s kappa. Each actor’s thematic agenda was displayed through colours in egocentric maps.

To analyse thematic cliques, we explored the distribution of thematic agendas of actors. We examined data to see if one of the thematic categories was over−/underrepresented in Upper Palatinate or Lower Bavaria.

To analyse geographical clusters and gaps, we created a graph that shows the number of actors per district or city not attached to a district with MS Power BI. For the analysis of data on the district level, data for Upper Palatinate and Lower Bavaria was combined (Upper Palatinate and Lower Bavaria consist of 22 districts in total). We used heat maps to reveal clusters in the networks.

To illustrate the context in which actors operate, we analysed associations between the distribution of actors, deprivation, and age. SAS software, release 9.04.01M6P11072018, was used for statistical analyses and visualisation. We plotted the number of actors per district against the BIMD to identify districts with the highest deprivation values (as compared to the other districts in this study) and districts without a health promotion actor. Furthermore, we plotted the number of actors against age distributions to identify districts with high ageing quotients and districts that remain unsupported by a health promotion actor. Pearson’s *r* was used to assess correlations between the BIMD and the ageing quotient in order to identify highly deprived areas with high ageing quotients. Pearson correlation coefficients with *p*-values ≤0.05 were considered statistically significant. We focused on the ageing quotient because results revealed a strong surplus of actors with the thematic agenda “healthy ageing” in Upper Palatinate, a network dynamic that had been developing independently after a highly successful event in this thematic field.

## 3. Results

### 3.1. Identification of health promotion actors

Through data preparation, 55 actors in Upper Palatinate were identified. The data collection yielded a data set for Lower Bavaria with 64 actors. Actors work for public health departments, associations or organisations, schools or universities, political institutions, and volunteer organisations. Further information about descriptive aspects and level of competence of actors can be found in the supplementary material.

### 3.2. Thematic agenda and frequency and strengths of contacts

**Figure 2:**
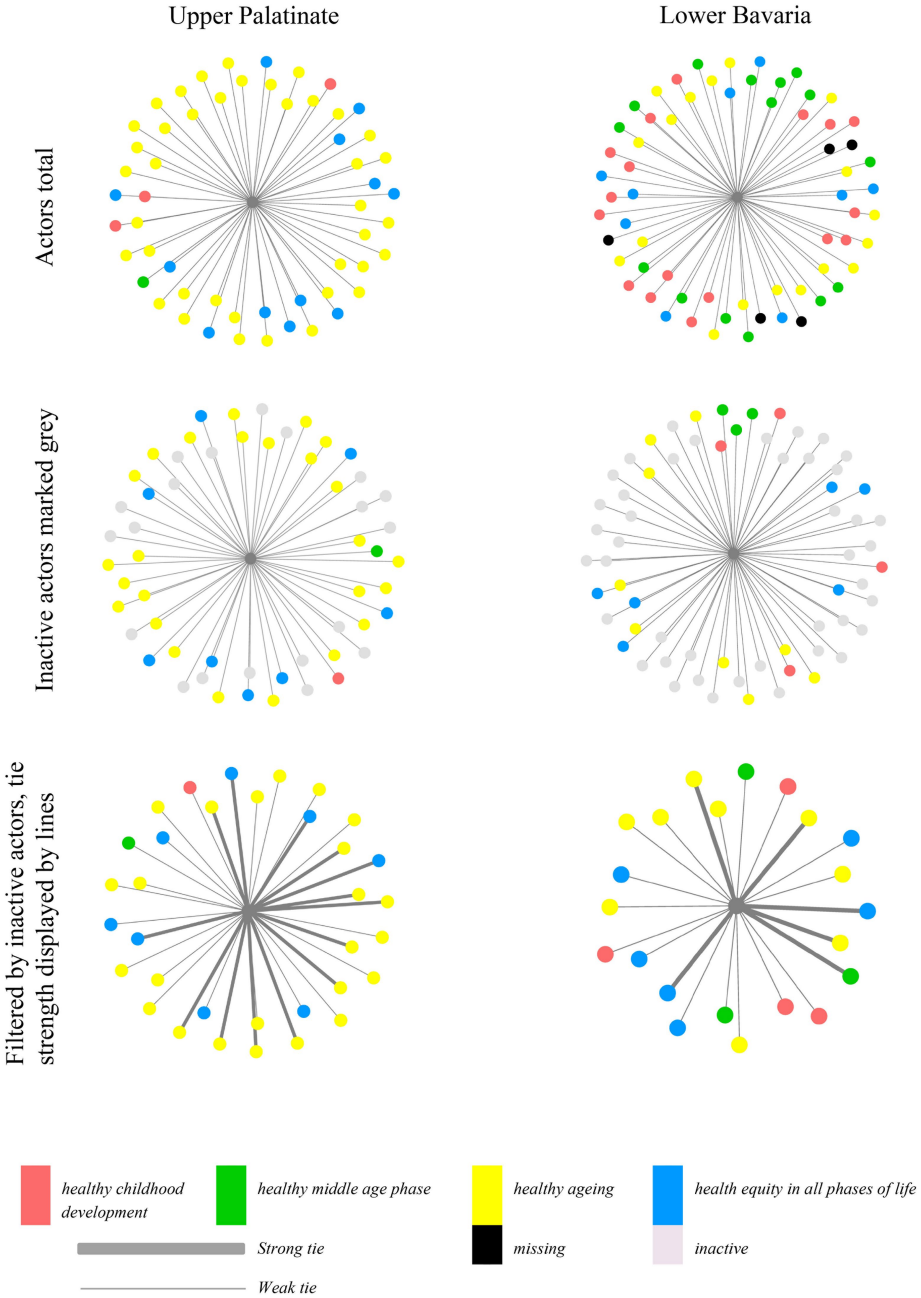
Actors in Upper Palatinate and Lower Bavaria coloured by thematic agenda. Outer nodes represent individual actors and their thematic agenda. Lines between the nodes illustrate a tie; the thickness of a line represents its strength. Inactive actors are coloured grey. In the third graph, inactive actors were excluded.

Results of the content analysis verified the pre-existing categories “healthy childhood development,” “healthy middle age phase,” “healthy ageing,” and “health equity in all phases of life” as the actors’ thematic agendas. The interrater reliability was high, with Cohen’s Kappa *r* = 0.81 (*p* < 0.0001) (23). Figure 2 shows egocentric maps of the networks. The thematic agenda is illustrated through the node’s colour. The KGC, the network’s initiator, is shown as the central node (illustrated through the middle node).

Upper Palatinate had more active ties than Lower Bavaria *(Upper Palatinate: n1(active ties) = 34; Lower Bavaria: n2(active ties) = 22)*, thereof n1 = 14 and n2 = 6 strong ties (meaning contact at least quarterly). In Upper Palatinate, 70.9% of actors focused on “healthy ageing” *(Lower Bavaria: n2(healthy ageing) = 39)*. Three actors belonged to the “healthy childhood development” field. Two of those actors are marked as inactive. One actor belonged to “healthy middle age phase*.”*

Distribution of the thematic agenda in Lower Bavaria was more equal *(“healthy ageing” n2 = 18, 28.1%; “healthy childhood development” n2 = 17, 26. 6%; “healthy middle age phase” n2 = 15, 23.4%; “health equity in all phases of life” n2 = 9, 14.1%).*

### 3.3. Geographical distribution and clusters

**Figure 3:**
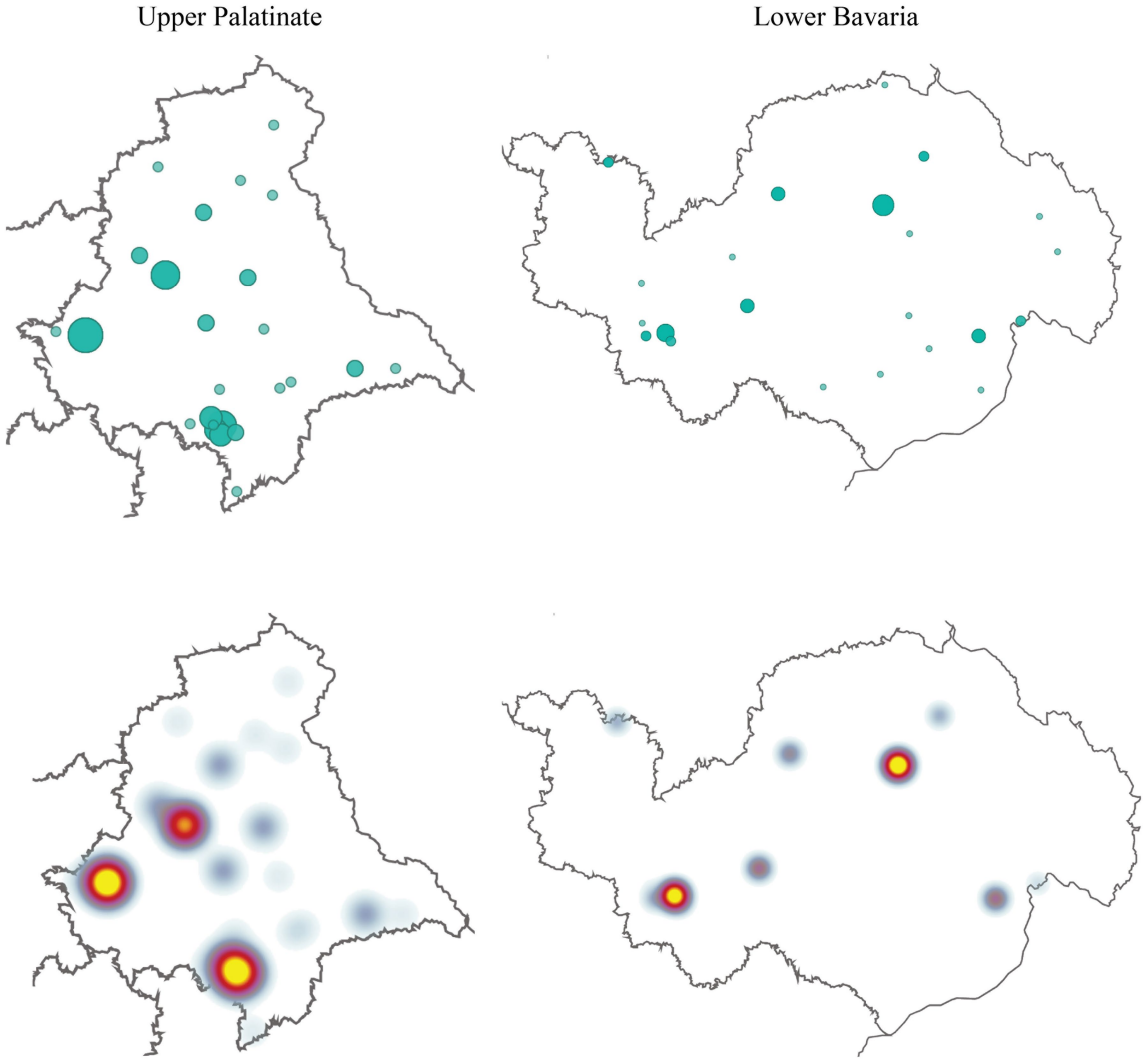
Geographical distribution of actors. Presented in geographical maps and heat maps. Larger green dots indicate actors with the same postal codes.

**Figure 4:**
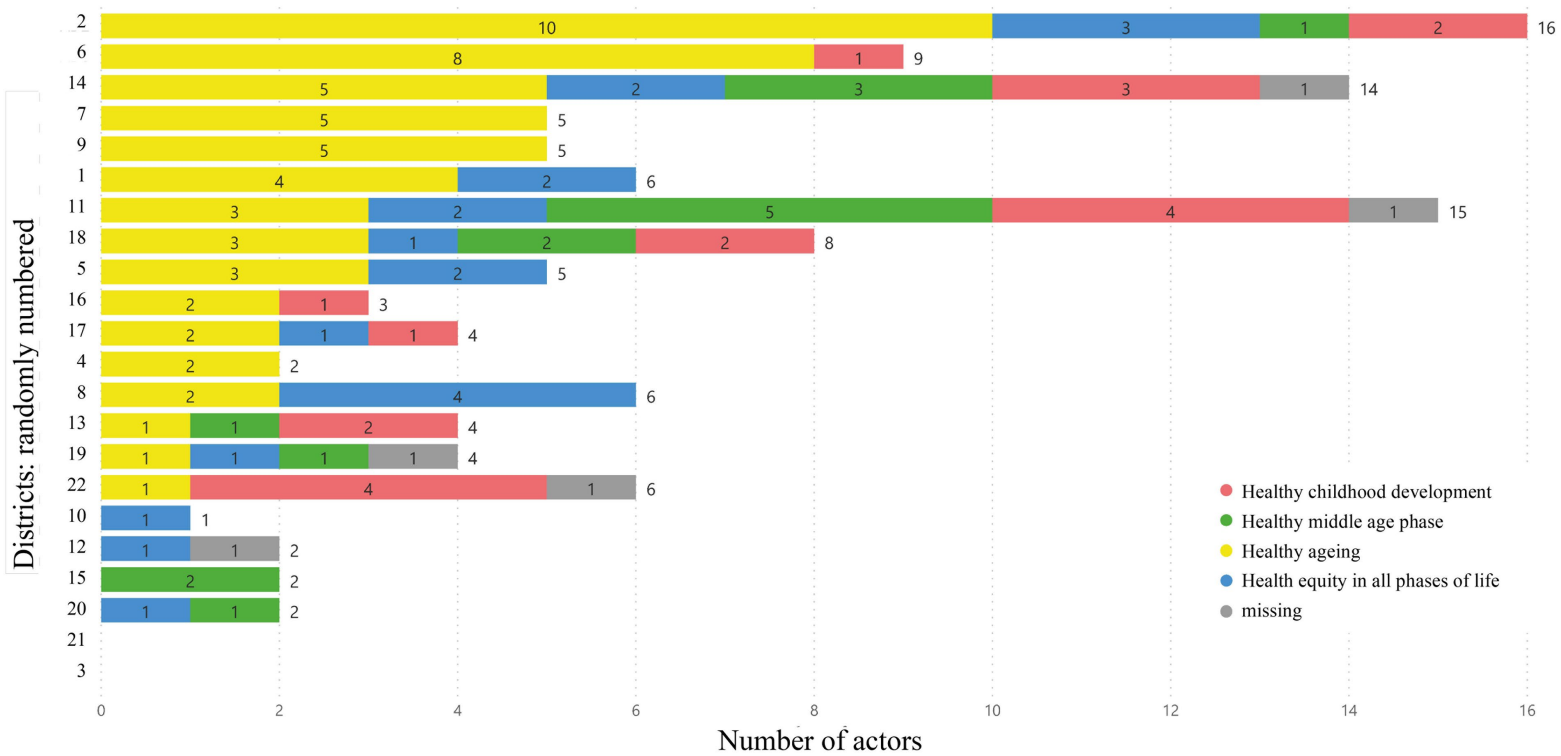
Geographical distribution of actors. Presented in a stacked bar chat. Graphs show the number of actors per district of Upper Palatinate and Lower Bavaria combined (divided into fields: “healthy childhood development”, “healthy middle age phase”, “healthy ageing”, “health equity in all phases of life”).

Three geographical clusters were identified in Upper Palatinate and two in Lower Bavaria. Results are presented as cartographical maps (Figure 3) and as a graph of the total number of actors per district (Figure 4). The graph shows a smaller number of clusters because some clusters consist of actors who are located in different districts, but which are still geographically proximal. Two districts with no health-promoting actors were identified, one of them close to the border of the Czech Republic.

### 3.4. Context analysis: deprivation index and ageing quotient

**Figure 5:**
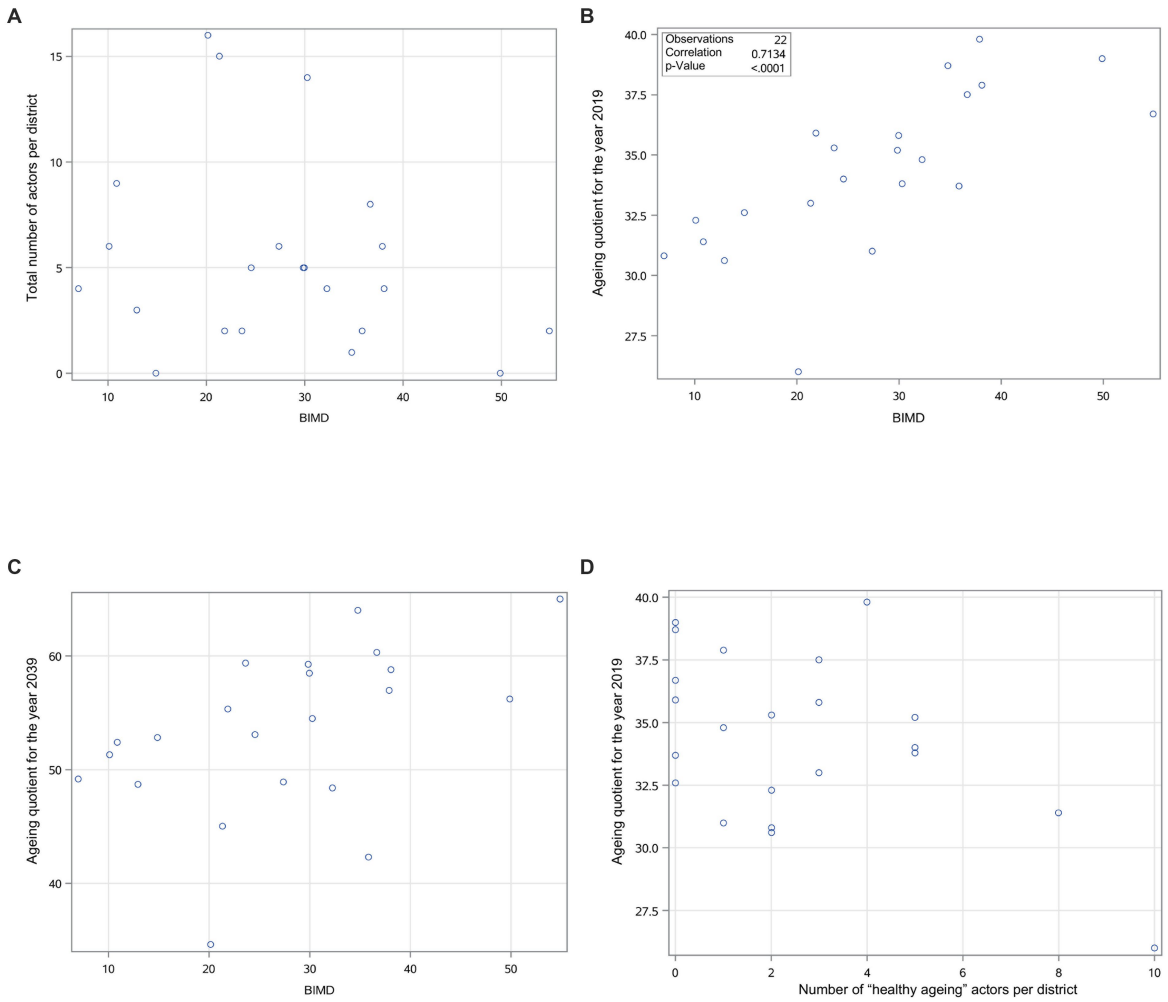
Scatter plots showing relationships between the BIMD and the total number of actors, the BIMD and the ageing quotient for the year 2019/2039, and the number of “healthy ageing” actors and the ageing quotient for 2019. Each dot represents one district in Upper Palatinate or Lower Bavaria.

Figure 5 presents scatter plots for the data set of Upper Palatinate and Lower Bavaria combined. Each dot represents one district that belongs to either Upper Palatinate or Lower Bavaria. In graphic (A) the total number of actors is plotted against the BIMD. This showcases differing BIMD values *(14.9, 49.9)* for the two districts with no health promotion actor. The highest BIMD value of all considered districts is 54.9. This district has two actors.

The second plot (B) illustrates the correlation of BIMD with the ageing quotient in 2019. The relationship shows a positive correlation *(r = 0.71; p < 0.0001)* of the BIMD with the ageing quotient. The two districts with the highest BIMD values *(54.9; 49.9)* also exhibit high ageing quotients *(36.7; 39.0)*. The third plot (C) illustrates the relationship between the BIMD and the predicted ageing quotient for the year 2039. Two statistical outliers were identified. The first one is the district with the highest BIMD value *(54.9)*. It is predicted to exhibit the highest ageing quotient by 2039 *(65.0)*. The second outlier is predicted to have an ageing quotient of 34.6 *(arithmetic mean* $\bar{y}$ *= 53.4)*, with a BIMD value of 20.2 *(* $\bar{x}$ *= 27.5)*.

Lastly, the number of “healthy ageing” actors per district was plotted against the ageing quotient (D). We chose this specific thematic agenda because it had the highest number of actors, which indicates a potential relationship. No statistically significant relationship can be observed. The district with the highest number of “healthy ageing” actors *(n = 10)* is the one with the lowest ageing quotient *(26.0)*. However, this district has the highest number of actors in general *(n = 16)*.

## 4. Discussion

In this study, two health promotion networks of the KGC were explored with a focus on thematic agenda and geographical location. The number of actors linked to the KGC varied only slightly between Upper Palatinate and Lower Bavaria. Results regarding the strength of relationships show a high number of weak ties in Lower Bavaria. The presentation of actors’ geographical location and their thematic agenda demonstrates a strong surplus of actors with the thematic agenda “healthy ageing” in Upper Palatinate. Only one active tie to an actor with the thematic agenda “healthy childhood development” and one to an actor with the thematic agenda “healthy middle age phase” were found in Upper Palatinate. Furthermore, a geographical gap, i.e., two districts with no health promotion actor, was explored. By including deprivation values and age distribution, we identified two districts with high BIMD values and high ageing quotients.

In the following paragraphs, our findings are discussed with regard to existing studies and literature. A higher number of ties with health promotion actors is not directly related to increased network efficiency (24). Researchers in public health collaboratives emphasize a focus on quality over quantity (24). Trust is seen as the actual “key to good collaboration” (24). According to research about public health collaboratives, a shared mission strengthens trust (24). Hence, the thematic agendas may have revealed structures of trust as well, as they share the same target group, given that actors do not follow a competitive mindset, for example regarding scarce resources. Actors that are geographically close to one another may also collaborate more often, as geographical location can influence collaboration (25). Existing evidence identifies no disadvantage, but instead opportunities, in a high number of weak ties, such as those we identified in Lower Bavaria (17). This is due to a greater range of resources and perspectives (17). Sociologist Mark Granovetter has even gone so far as to advise network coordinators to increase their number of weak ties without reducing the number of strong ties in the process (17). Based on this, researchers concluded that strengthening ties to actors was most valuable for achieving a defined goal and for diffusing information (24).

Network cliques, such as those indicated by the surplus of “healthy ageing” actors found in Upper Palatinate, have both advantages and disadvantages. On the one hand, cooperation at clique level rather than across whole networks can increase effectiveness (26). On the other hand, cliques potentially lead to segregation (27). It should be mentioned that in network research, cliques imply that each member holds direct connections to all others (26). This was not a focus of our study. Nevertheless, we still find it important to compare our findings to existing literature regarding cliques. Cliques can also be located by identifying groups with the same interests, activities, or goals (28). Although we did not measure the actors` interests, activities, or goals, we hypothesize that actors with the same agenda potentially share activities or goals, for example joining the same event regarding a specific topic. Since only one active connection each to the thematic agendas “healthy childhood development” and “healthy middle age phase” were found in Upper Palatinate, results are more in line with findings about potential isolation (27).

Our contextualization of geographical gaps using analyses of BIMD values and ageing quotients can be compared to a recent study which geographically mapped network members and public awareness scores about child maltreatment (10). Using this process, underrepresented areas that might need further engagement were identified (10). In our study, data regarding BIMD and ageing quotients also helped to identify such areas. This can be seen in the following example: if two districts remain unsupported by actors, and one is more deprived than the other, it may be reasonable to focus on the more deprived district. This is because correlations between deprivation and the prevalence of diseases have been repeatedly discovered (29, 30). Additionally, if one district exhibits a high ageing quotient, it might be helpful to implement actors addressing the thematic agenda “healthy ageing.”

### 4.1. Limitations and strengths

Our study has some limitations. Inactive actors were not distinguished from active ones in the geographical analysis and in scatter plots. However, potentially weak ties were shown in separate figures (egocentric maps) to show this characteristic of the ties and minimize bias. Since deprivation was analysed at district level, deduction about individual needs is limited. Further research should map additional variables like resources given and assets, or existing programs and activities in health promotion. In addition, generalisability is limited, as our findings focus on a specific region and organisation. However, our findings can be applied to circumstances of other regions and organisations by measuring and analysing variables using the same methods. Another limitation is, that we excluded actors if they were located outside Upper Palatinate or Lower Bavaria, or if the frequency of contact was missing. If the excluded actors differ in important ways from the included actors, this can potentially lead to biased results. However, the authors expect the number of such actors to be minimal, since data was not only collected based on existing datasets and documents, but also through an expert survey. Hence, the sample should still be sufficient to obtain meaningful and unbiased results.

One strength of our methods helped to prevent a recall bias: the collection of data with a structuring content analysis. Experts did not have to list actors from memory and recall bias was avoided (5). Another benefit was the coding approach of the content analysis (22). This allowed us to calculate the interrater reliability and assure consistency of the answers of experts and the coding manual.

The strength of this study is the combination of evidence-based research and the implications for practice. This has resulted in a concept that allows data to be collected by the organisation itself without having to consult other network members. The current state of the data can be updated easily. This compensates for one disadvantage of SNA – the constant re-measurement of relationships between all actors (31). Such frequent data collection incurs high expenditures in terms of time and staff. In contrast, our approach allows an organisation to easily and quickly update data and figures, thus making it practical and sustainable to evaluate the network’s development.

### 4.2. Implications for practical work

Based on our findings, we suggest the following strategies to enable sustainable and effective networking for institutions like the KGC. First of all, we suggest strengthening trust by including trust-building activities to support effective collaboration within the network. Further, institutions could identify the most essential actors to achieve a defined goal, to strengthen relationships with them, and to disseminate information. For example, if an actor who supports the health of the older adults and is frequently in contact with an institution like the KGC, the institution can provide this actor with relevant information, as the actor is likely to spread it within his or her own network. A practical approach to expanding collaboration would also be to understand which actors are geographically close to one another and which actors share the same thematic agenda or target group. In this way, institutions can gain insight into potential clusters and gaps in the network and arrange future actions based on these insights. An institution could also study existing thematic agendas within their network in a more nuanced way and adapt the categorisation accordingly. This could lead to more detailed research findings, since a more in-depth study of existing thematic agendas could provide even more valuable insights into existing cliques and gaps within health promotion networks. Furthermore, institutions can prevent segregation of cliques by monitoring and managing ties between actors and underrepresented cliques closely. Finally, with the help of contextual information, institutions can align expenditures of the networks with the needs of a targeted area. Given our findings, institutions should focus on areas with high BIMD values and encourage determining the concentration of actors that serve each target group according to the existing age distribution.

## 5. Conclusion

By mapping health promotion networks thematically and geographically, this study offers a unique perspective to characterize such networks. Our results provide insight into structures of networks, thematic cliques, and geographical gaps. Hence, this new conceptualisation of mapping fits into the larger process of improving networks in the field of health equity and health promotion. Since possible strategies were derived from the mapping results, the findings contribute to broadening the range of mapping methods used and encourage further development of this methodology. Our study has the potential to improve decision-making processes concerning partnership strategies. Organisations such as the KGC can use this approach as a data-based starting point that leads to discussion and proposals for actions with a positive impact on health.

## Data availability statement

The raw data supporting the conclusions of this article will be made available by the authors, without undue reservation.

## Author contributions

The idea for this study came from AW. AF, KS, MC, and AW designed the study. AF collected the data and planned and performed the statistical analysis with the help of MC and KS and wrote the first draft of the manuscript. All authors contributed to the article and approved the submitted version.

## Funding

The work of the authors AW and KS is funded by the Federal Center of Health Education (BZgA) in the context of the GKV Alliance for Health (www.gkv-buendnis.de, accessed on 25 October 2022). The funder played no role in study design, data collection and analysis, decision to publish, and preparation of the manuscript.

## Conflict of interest

The authors declare that the research was conducted in the absence of any commercial or financial relationships that could be construed as a potential conflict of interest.

## Publisher’s note

All claims expressed in this article are solely those of the authors and do not necessarily represent those of their affiliated organizations, or those of the publisher, the editors and the reviewers. Any product that may be evaluated in this article, or claim that may be made by its manufacturer, is not guaranteed or endorsed by the publisher.

## Footnotes

^1^Bavarian Association for Health Promotion and Disease Prevention. Coordinating Office for Health Equity [cited 2022 Jun 23]. Available from: URL: https://lzg-bayern.de/fields-of-action/coordinating-office-for-health-equity.

^2^Landeszentrale für Gesundheit in Bayern e. V. Landeszentrale für Gesundheit in Bayern e. V. [cited 2022 Jun 23]. Available from: URL: https://lzg-bayern.de/.
